# Genetic variation at the CD28 locus and its impact on expansion of pro-inflammatory CD28 negative T cells in healthy individuals

**DOI:** 10.1038/s41598-017-07967-2

**Published:** 2017-08-09

**Authors:** Evaggelia Liaskou, Louisa Jeffery, Dimitrios Chanouzas, Blagoje Soskic, Michael F. Seldin, Lorraine Harper, David Sansom, Gideon M. Hirschfield

**Affiliations:** 1 Centre for Liver Research and NIHR Birmingham Liver Biomedical Research Centre, Institute of Immunology and Immunotherapy, University of Birmingham, Birmingham, UK; 2 Centre for Rare Diseases, Institute of Translational Medicine, Birmingham Health Partners, University Hospitals Birmingham, Birmingham, UK; 3 Institute of Metabolism and Systems Research, College of Medical and Dental Sciences, University of Birmingham, Birmingham, UK; 4 Institute of Inflammation and Ageing, University of Birmingham, Birmingham, UK; 5 Institute of Immunity and Transplantation, University College London and Royal Free Hospital, London, NW3 2PF UK; 6 Department of Biochemistry and Molecular Medicine, University of California at Davis, Davis, CA 95616 USA; 7 Division of Rheumatology, Allergy and Clinical Immunology, University of California at Davis School of Medicine, Genome and Biomedical Sciences Facility, 451 Health Sciences Drive, Suite 6510, Davis, CA 95616 USA

## Abstract

The *CD28* locus is associated with susceptibility to a variety of autoimmune and immune-mediated inflammatory diseases including primary sclerosing cholangitis (PSC). Previously, we linked the CD28 pathway in PSC disease pathology and found that vitamin D could maintain CD28 expression. Here, we assessed whether the PSC-associated *CD28* risk variant A (rs7426056) affects CD28 expression and T cell function in healthy individuals (n = 14 AA, n = 14 AG, n = 14 GG). Homozygotes for the PSC disease risk allele (AA) showed significantly lower *CD28* mRNA expression *ex-vivo* than either GG or AG (*p* < 0.001) in total peripheral blood mononuclear cells. However, the *CD28* risk variant alone was not sufficient to explain CD28 protein loss on CD4^+^ T cells. All genotypes responded equally to vitamin D as indicated by induction of a regulatory phenotype and an increased anti-inflammatory/pro-inflammatory cytokine ratio. A genotypic effect on response to TNFα stimuli was detected, which was inhibited by vitamin D. Together our results show: (a) an altered gene expression in carriers of the susceptible CD28 variant, (b) no differences in protein levels on CD4^+^ T cells, and (c) a protective effect of the variant upon CD28 protein loss on CD4^+^ T cells under inflammatory conditions.

## Introduction

Primary sclerosing cholangitis (PSC) is a poorly understood chronic immune-mediated liver disease represented by widespread fibrotic strictures of the intra- and the extra-hepatic biliary tree. PSC is a devastating disease that lacks effective treatment and validated animal models. To date, several risk loci have been identified for PSC, with the large majority of them involving genes encoding molecules that serve essential functions in immune-related pathways^1^.

**Figure 1:**
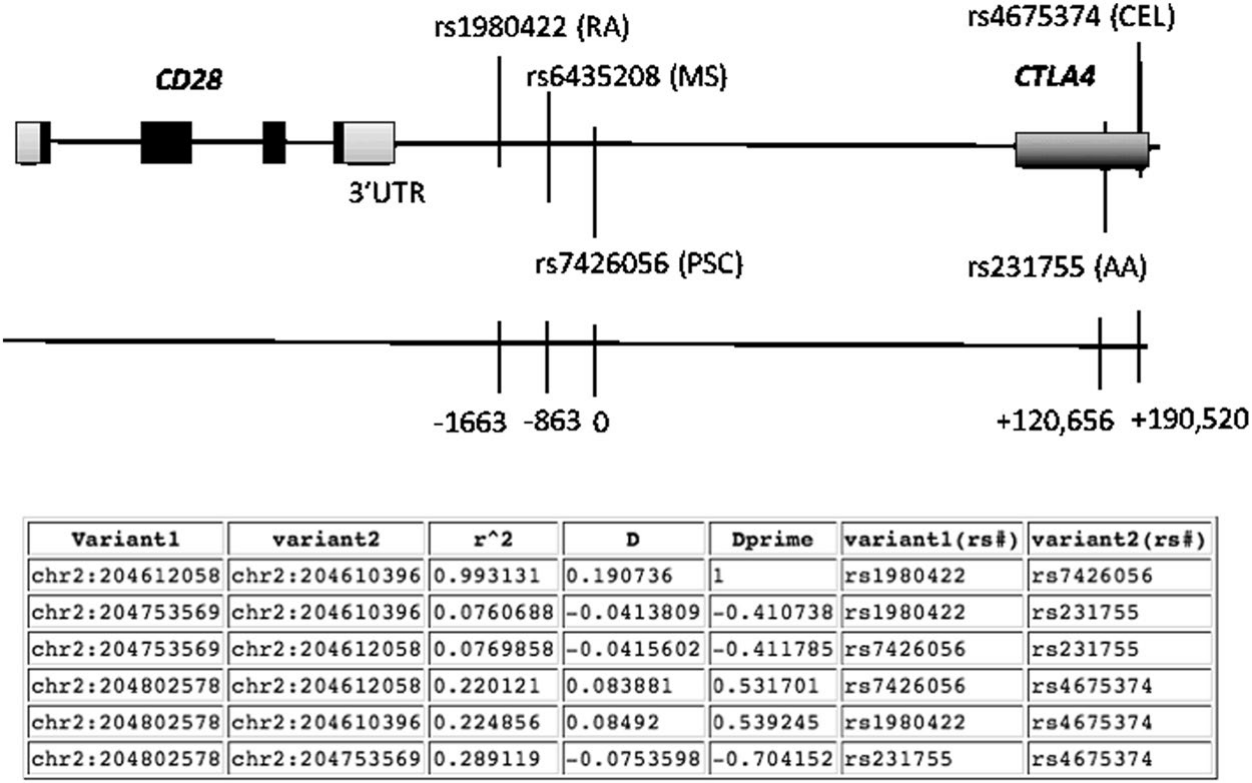
Location of rs7426056 single nucleotide polymorphism on *CD28* locus. Rs7426056 SNP is located between *CD28* and *CTLA4* genes; approximately 3.5 kb downstream the CD28 3′UTR and approximately 120 kb upstream *CTLA4* gene. Several risk variants in the *CD28/CTLA4* locus have been associated with other autoimmune and immune-mediated diseases. Exons are indicated in black. (**B**) Table shows the linkage disequilibrium of rs7426056 with the other SNPs in *CD28* and *CTLA4* genes. PSC: primary sclerosing cholangitis, RA: rheumatoid arthritis, MS: multiple sclerosis, AA: alopecia areata, CEL: celiac disease.

The *CD28* locus is a newly recognized risk factor in PSC development^2, 3^; different genetic variants within the *CD28/CTLA4* locus have been also associated with rheumatoid arthritis^4^, celiac disease^5^, alopecia areata^6^ and more recently with multiple sclerosis^7^ (an overview of the different SNPs and their location in relation to PSC risk variant is shown in Fig. 1). Because the CD28 protein is an important co-stimulatory molecule involved in the survival, clonal expansion, IL-2 production and metabolic activity of T cells^8^, it is predicted that such variants of CD28 will have functional impact on immune activation. From studies in several inflammatory diseases, including PSC, it is evident that the CD28 pathway has relevance to disease biology^3^_._ However, thus far, the biological implications of such variants are not clear, limiting translation of genetic discoveries through to biologic impact.

The genetic variant “rs7426056” in the *CD28* gene locus associated with PSC (minor allele A) is sufficiently common (0.229 in controls) to facilitate investigation in human lymphocytes^1^. Therefore, to probe our hypothesis that there are functional differences related to CD28 expression and function based on genetic background, we studied healthy subjects genotyped for this CD28 risk variant, evaluating: (a) basal CD28 expression and (b) phenotype and function of activated CD4^+^ T cells.

## Results

### CD28 mRNA expression is genotype dependent

**Figure 2:**
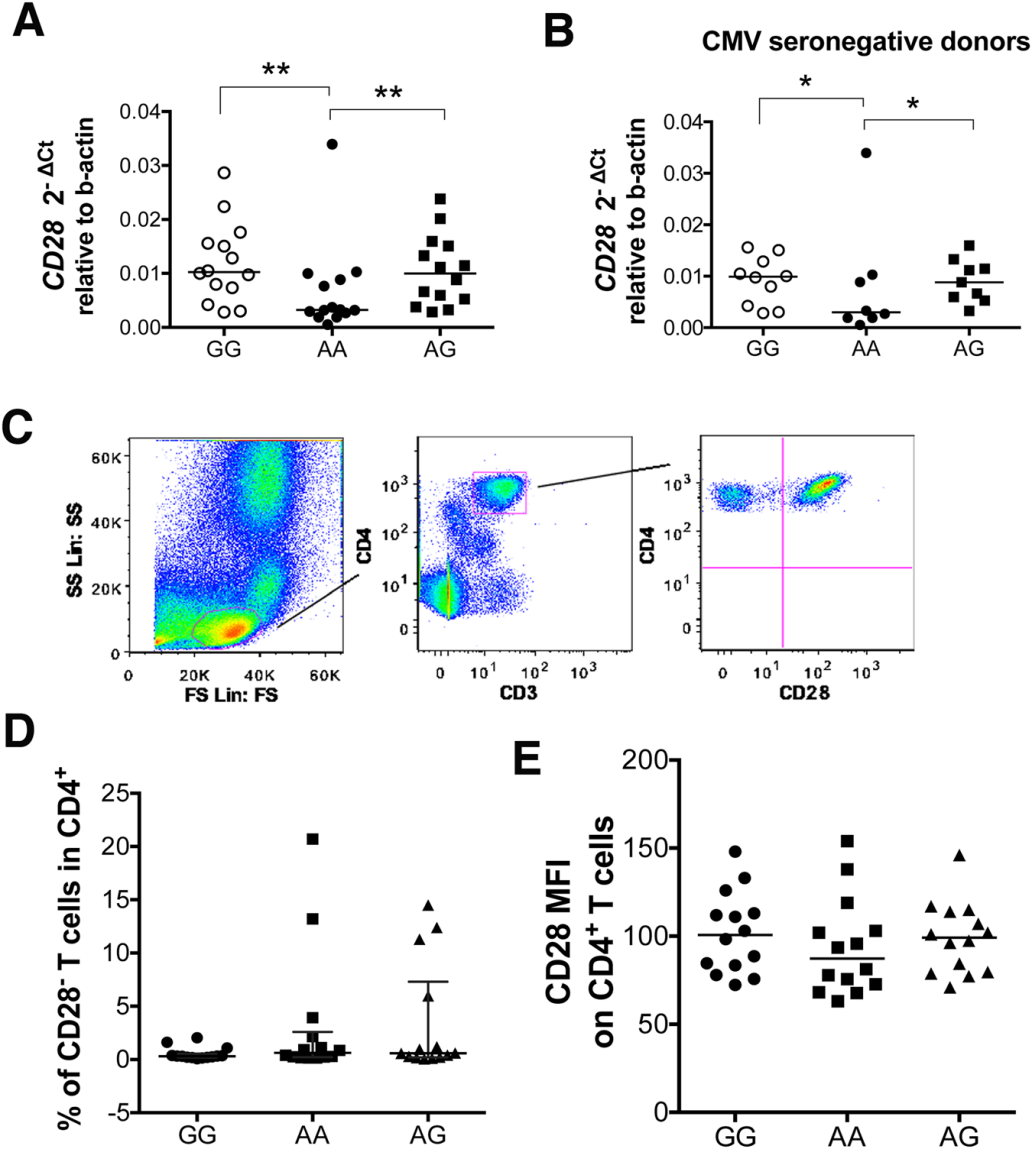
AA subjects show reduced CD28 mRNA expression but similar frequencies of CD4^+^CD28^−^ T cells with GG and AG. (**A**) RNA from freshly isolated peripheral blood mononuclear cells (PBMCs) was extracted, transcribed into cDNA and tested for CD28 mRNA expression in healthy individuals homozygous (AA) or heterozygous (AG) for the PSC risk variant and homozygous for the protective allele (GG) (n = 14 per group). Data are expressed as 2^−ΔCt^ relative to beta actin. Lines indicate median values. **p = 0.002 and **p = 0.003 with Mann Whitney U test after exclusion of the outlier detected in the AA group by Grubbs test. (**B**) *CD28* mRNA expression in total PBMCs in CMV seronegative donors. Data are expressed as 2^−ΔCt^ relative to beta actin. AA and AG healthy individuals homozygotes and heterozygotes for the PSC risk variant and GG homozygotes for the protective allele. Lines indicate median values. *p = 0.02 and *p = 0.03 with Mann Whitney U test after exclusion of the outlier detected in the AA group by Grubbs test. (**C**) Representative flow cytometry plots showing the gating strategy to define CD4^+^CD28^−^ T cells in PBMCs. CD3^+^CD4^+^ cells were selected after duplet exclusion and CD28 expression was studied in the CD4^+^ population. (**D**) Data show the proportion of CD4^+^ T cells that have lost CD28 expression. Line indicates median with interquartile range. (**E**) CD28 median fluorescence intensity (MFI) across genotypes. Line indicates median value.

The gender and age of all subjects was equal between groups [GG: 45 (range: 32–53 years), AA: 47.5 (range: 37–57 years), and AG: 45.5 (range: 33–53). *CD28* mRNA expression was significantly lower in AA (2^−ΔCt^ = 0.003) compared to GG (0.01, *p* < 0.001) and AG (0.009, *p* < 0.001) subjects when studying total peripheral blood mononuclear cells (see Methods) (Fig. 2A). However, we did not detect a statistically significant difference in peripheral blood CD4^+^CD28^−^ T cell frequencies between the different genotypes (Fig. 2C,D). Studying CD28 cell surface MFI also revealed no statistically significant difference across genotypes [GG: median = 101 (range: 72–148), AA: 87 (63–154), AG: 99 (71–146)] (Fig. 2E). A positive correlation between frequency of CD4^+^CD28^−^ T cells and CMV positivity was observed (Supplementary Figure 1A), as previously described^9^. After exclusion of CMV seropositive donors from the analysis, however, AA carriers still showed reduced CD28 mRNA expression (0.0029 median value) compared to GG (0.0098) and AG (0.0088) (Fig. 1B). Grubbs test analysis or Box plot revealed the presence of one extreme outlier in the AA group (value of 2^−ΔCt^ of 0.03) and its removal from the statistical analysis showed that the CD28 mRNA expression levels in AA were significantly lower compared to GG (p = 0.02) and AG (p = 0.03). CD28 is expressed by both CD4 and CD8 T cells; mRNA expression was studied in total PBMCs thus the *CD28* expression levels should be also attributed to CD8^+^ T cells. In CD8^+^ T cells no statistically significant differences in frequency of CD28^−^ T cells was detected between the different genotypes (Supplementary Figure 1B). Exclusion of CMV seropositive donors when studying CD28 protein expression and specifically the frequency of CD28^−^ T cells in the CD4 and CD8 population results in similar frequencies across genotypes [% of CD28^−^ T cells in CD4 in GG: median = 0.279 (range 0.08–0.37), AA: 0.272 (0.15–1.13), AG: 0.294 (0.06–1.2)] [% of CD28^−^ T cells in CD8 in GG: median = 21.1 (range 3.83–69.4), AA: 15.75 (8.63–54), AG: 18 (12.6–47.2)] (Supplementary Figure 1C,D). These data show that CMV seropositive donors have higher frequencies of CD28^−^ T cells. However, it is noteworthy that in GG individuals only 3/13 (23%) were CMV seropositive, whereas in AA and AG 5/13 (38%) and 4/13 (31%), respectively, were CMV seropositive. Collectively, our data suggest that an interplay between viral infection and genotype might be important for CD28 expression.

### The *CD28* risk variant alone is not sufficient to explain CD28 loss

We have recently reported that CD4^+^CD28^−^ T cells accumulate in livers of patients with PSC, where they localize close to the bile ducts and can induce the death and activation of the latter^3^. High levels of TNFα were detected in the liver of PSC patients and we showed that TNFα could downregulate the expression of CD28 on PSC T cells *in vitro* ^3^_._ CD4^+^CD28^−^ T cells serve as a surrogate marker of inflammation in several autoimmune and immune-mediated inflammatory diseases^10–12^, therefore we assessed whether there are biologic differences across the genotypes in the context of CD4^+^CD28^−^ T cell expansion after TNFα stimulation.

**Figure 3:**
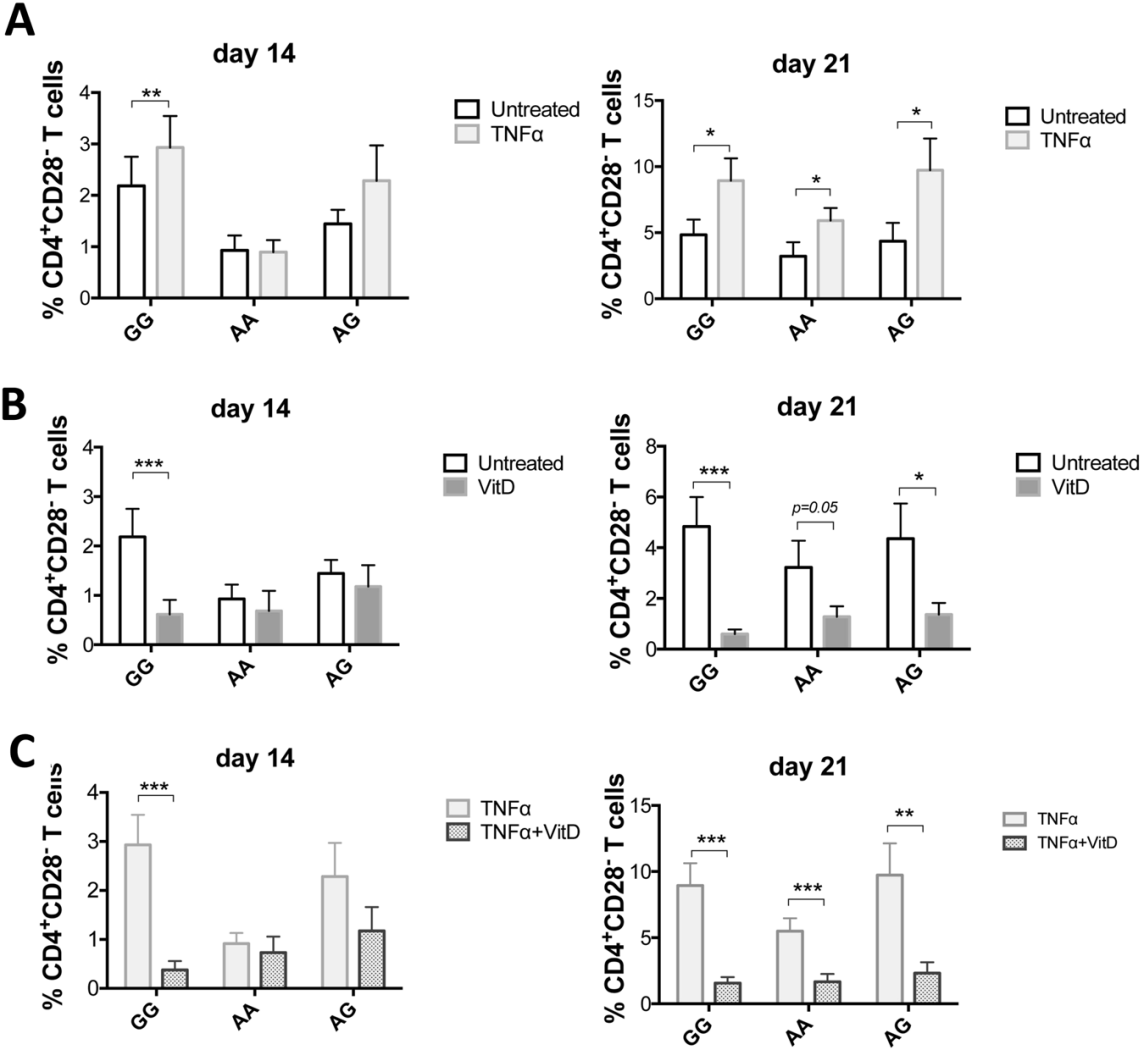
Individuals with AA and AG genotypes are less susceptible to TNFα-mediated loss of CD28 expression. CD4^+^CD25^−^ T cells were activated with aCD3/aCD28 beads (two cycles of activation) in the presence or absence of TNFα and in the presence or absence of 1,25(OH)_2_D_3_ for 14 and 21 days. (**A** and **B**) Data show the proportion of CD4^+^CD25^−^ T cells that lost CD28 expression in the presence (TNFα) and absence of TNFα (Untreated) and in the presence or absence of 1,25(OH)_2_D_3_ after 14 and 21 days. (**C**) Data show the proportion of CD4^+^CD25^−^ T cells that lost CD28 expression in the presence of TNFα and in the simultaneous presence of TNFα and 1,25(OH)_2_D_3_ after 14 and 21 days in culture. Healthy individuals homozygous (AA) (n = 14) or heterozygous (AG) (n = 14) for the PSC risk SNP “rs7426056” in the CD28 locus and n = 14 homozygous for the protective allele (GG). Data show mean with SEM. *p < 0.05, **p < 0.01, ***p < 0.001 with Wilcoxon matched-pairs signed rank test.

After 21 days TNFα enhanced the expansion of CD4^+^CD28^−^ T cells across all genotypes. This effect was not significantly different at day 14 for AA and AG genotypes, in keeping with a delayed response to TNFα-mediated loss of CD28 expression in at risk subjects (Fig. 3A). Even in untreated conditions where only two cycles of TCR stimulation were applied in the absence of TNFα, in cells with the GG genotype there was a greater expansion of CD4^+^CD28^−^ T cells at day 14 compared to AA and AG (Fig. 3A).

### Carriers of the wild type allele (GG) are more susceptible to the pro-inflammatory effects of TNFα but also responsive to the protective effect of vitamin D

We have previously reported that active vitamin D (1,25(OH)_2_D_3_) can overcome the effect of TNFα upon loss of CD28 expression^3^, therefore we studied whether there are genotype-associated differences in the ability of T cells to respond to 1,25(OH)_2_D_3_ vitamin D. In GG, 1,25(OH)_2_D_3_ significantly reduced the frequency of CD4^+^CD28^−^ T cells in the presence of TNFα at both day 14 and day 21 (5- and 4.3- fold, respectively, *p* < 0.001). In AA and AG, this effect was significant only at day 21 (2.5-fold mean suppression in both cases, *p* < 0.001, *p* < 0.01) (Fig. 3B and C), possibly because at day 14 there was not a significant induction of CD4^+^CD28^−^ T cells by TNFα. Collectively, our data indicate that carriers of the wild type allele (GG) are more susceptible to the pro-inflammatory effects of TNFα but also remain responsive to the protective effect of vitamin D.

### T cells from the different genotypes are equally responsive to 1,25(OH)_2_D_3_

We employed a system with fixed CHO-CD80 cells to stimulate CD4^+^CD25^−^ T cells and study the ability of the latter to respond (by cytokine production, up-regulation of activation markers and proliferation) as this system has been well characterised in former published studies performed by our collaborators^13, 14^. These studies demonstrate the necessity of the CHO-CD80 co-stimulatory signal for T cell proliferation, even in the presence of anti-CD3, and its stronger ability to promote T cell proliferation compared to CD86, consistent with the greater affinity of CD80 for CD28 compared to CD86. Former studies have also shown the ability of the ligand binding domain of CTLA-4 (CTLA-4-Ig) to block the fixed CD80 signal provided by fixed CHO-CD80 cells, preventing T cell proliferation^14^.

**Figure 4:**
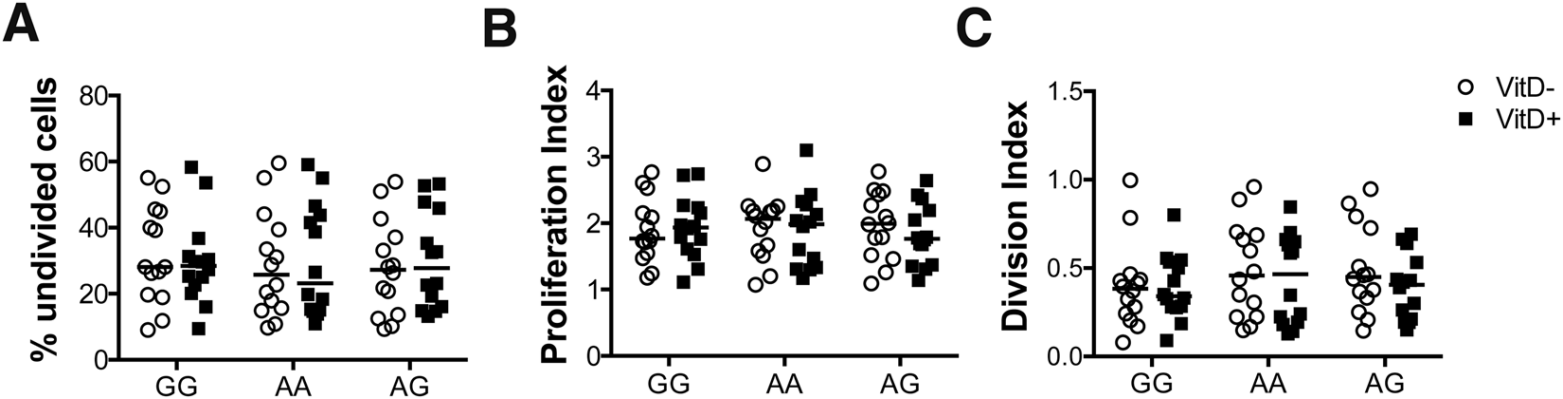
GG, AA and AG individuals show similar levels of proliferation. CD4^+^CD25^−^ T cells were activated *in vitro* with aCD3 antibody and CHO-CD80 cell line in the presence or absence of 1,25(OH)_2_D_3_ for 5 days. Data show (**A**) the percentage of undivided cells, (**B**) the proliferation index and (**C**) the division index across genotypes in the presence (VitD+) and absence (VitD−) of vitamin D.

T cell activation and CD28 signaling promote T cell proliferation, therefore we assessed whether the T cells from subjects with the risk variant show impaired proliferation capacity. Our data show that T cells from all genotypes were equally able to proliferate as indicated by the similar percentage of undivided cells [GG: median = 28.1% (range: 9.03–55.1), AA: median = 25.8% (range: 9.69–59.6), AG: median = 27.2% (range: 9.28–53.9)]. Treatment with vitamin D did not alter this frequency, which was again similar across genotypes [GG: median = 28.45% (range: 9.44–58.3), AA: median = 23.15% (range: 10.9–59.1), AG: median = 27.8% (range: 13.2–53.2)] (Fig. 4A). Evaluation of the proliferation index, which shows the average number of divisions for those cells that went into division, showed similar values across genotypes in the presence and absence of vitamin D (Fig. 4B). To account for cells that never divided we further calculated the division index, a measure of the average number of divisions that a cell in the original population has undergone. This was likewise similar across genotypes in the presence and absence of vitamin D (Fig. 4C).

**Figure 5:**
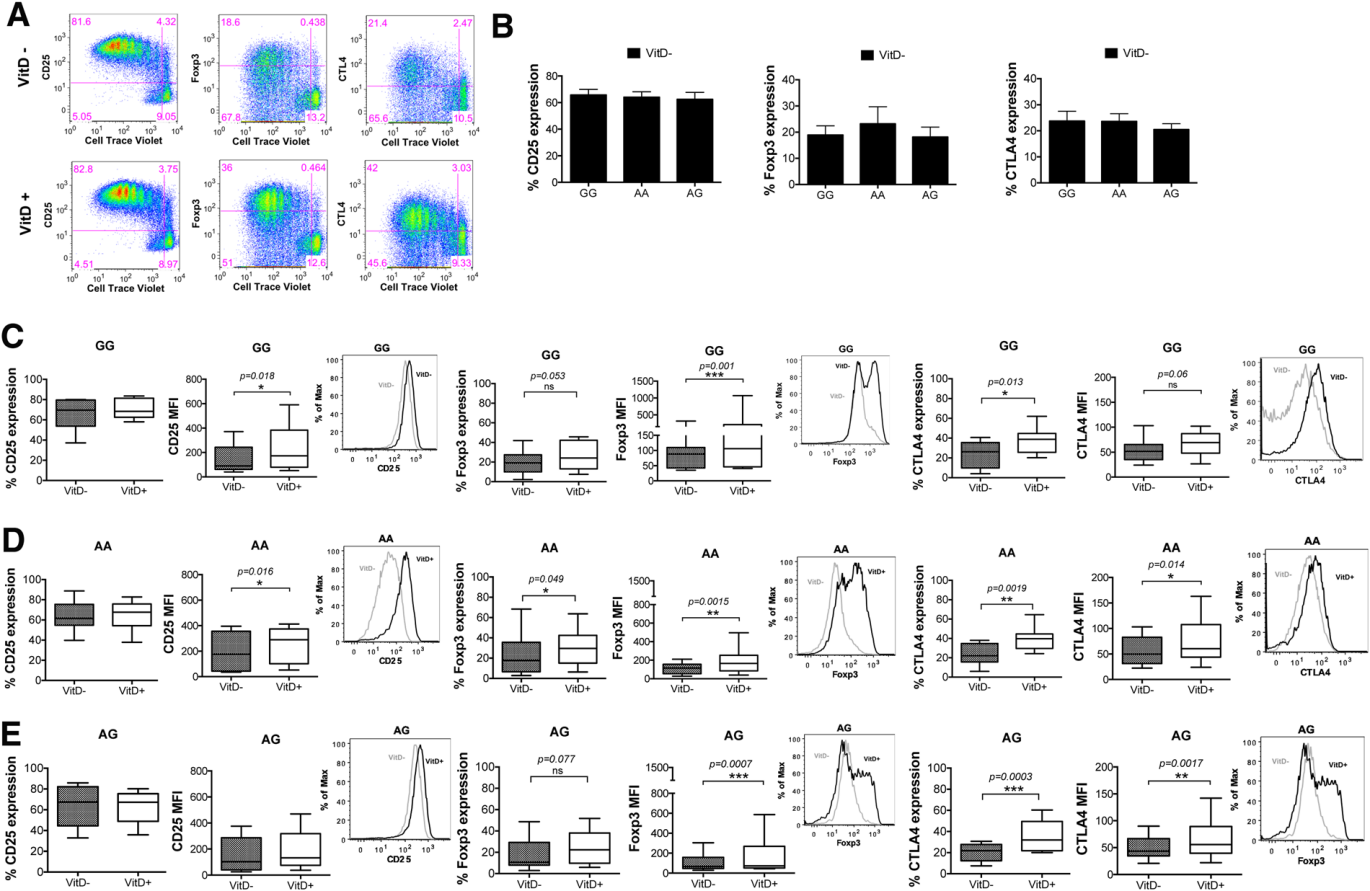
AA, AG and GG individuals express similar levels of CD25, Foxp3 and CTLA-4 molecules. CD4^+^CD25^−^ T cells were activated *in vitro* with aCD3 antibody and CHO-CD80 cell line in the presence or absence of 1,25(OH)_2_D_3_ for 5 days. (**A**) Representative flow cytometry plots showing proliferating T cells expressing CD25, Foxp3 and CTLA-4. (**B**) Percentage of proliferating cells that express CD25, Foxp3 and CTLA-4 under no vitamin D treatment conditions (VitD−). (**C–E**) Data show the percentage of proliferating cells that express CD25, Foxp3 and CTLA-4 and their respective median fluorescence intensity values for GG, AA and AG genotypes of the PSC CD28 risk allele locus. Representative overlay histograms showing the expression of each marker in the presence and absence of 1,25(OH)_2_D_3_. *p < 0.05, **p < 0.01, ***p < 0.001 using Wilcoxon matched-pairs signed rank test.

**Figure 6:**
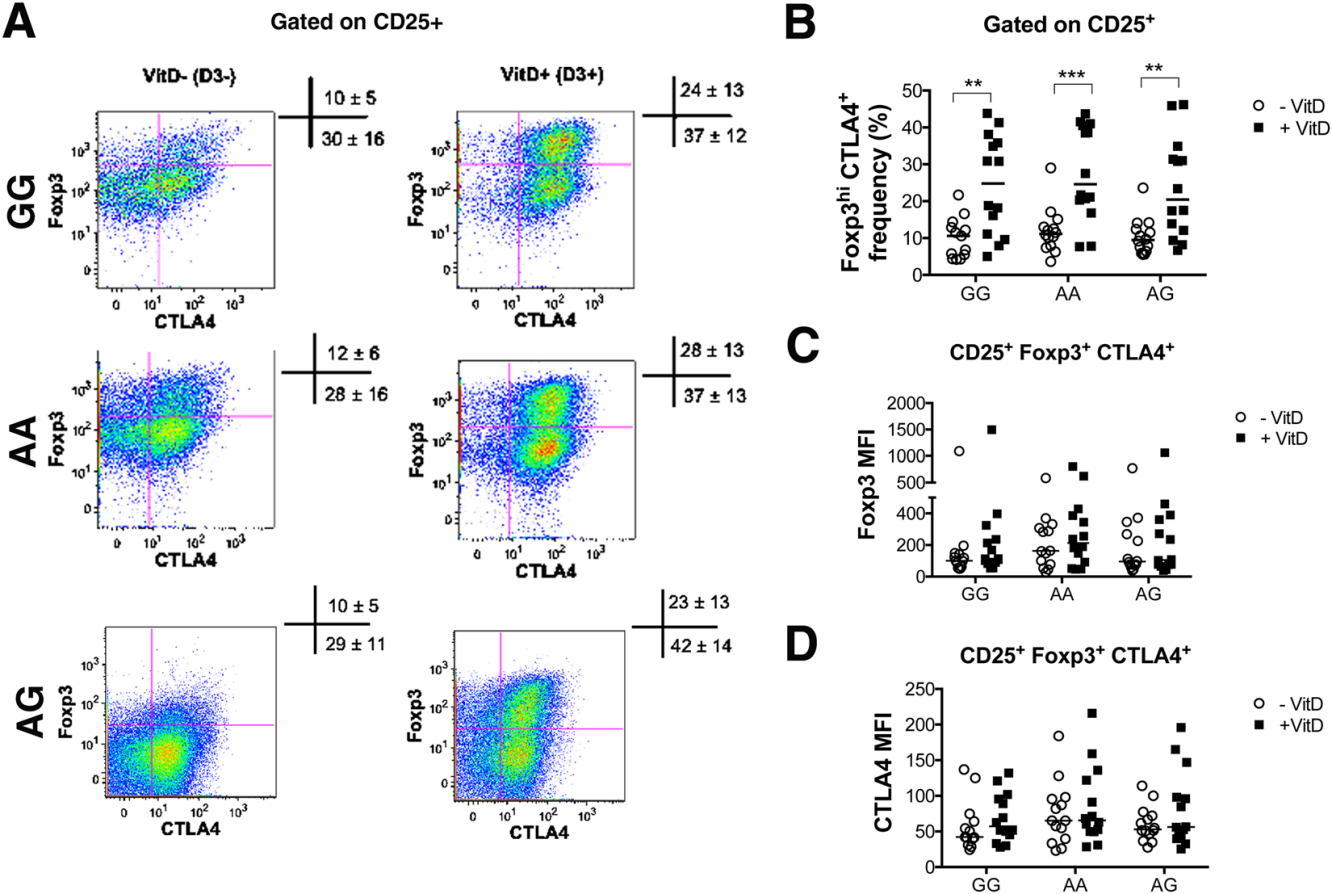
GG, AA and AG individuals show similar frequencies of Tregs. CD4^+^CD25^−^ T cells were activated *in vitro* with aCD3 antibody and CHO-CD80 cell line in the presence or absence of 1,25(OH)_2_D_3_ for 5 days. (**A**) Representative flow cytometry plots showing expression of Foxp3 and CTLA4 on CD25^+^ cells across genotypes in the presence (VitD+) and absence (VitD−) of 1,25(OH)_2_D_3_. Values in cross symbols represent the mean frequency (%) of Foxp3^hi^ CTLA4^+^ and Foxp3^int^ CTLA4^+^ plus/minus standard deviation across genotypes in all samples (n = 14 per group). (**B**) Frequency of Foxp3^hi^ CTLA4^+^ in CD25^+^ cells in the presence and absence of vitamin D. (**C,D**) Foxp3 and CTLA4 median fluorescence intensity (MFI) on Foxp3^hi^ CTLA4^+^ cells.

T cell activation and CD28 signaling also promote the expression of Foxp3, CD25 and CTLA-4 molecules^15^, therefore we assessed whether the T cells from subjects with the risk variant are functionally defective in their CD28 signaling. CD4^+^CD25^−^ T cells were stimulated for 5 days with anti-CD3 (aCD3) in the presence of CD80 expressed on CHO cells and the expression of the aforementioned markers was assessed by flow cytometry. No significant differences were detected between genotypes (Fig. 5). 1,25(OH)_2_D_3_ vitamin D has predominantly an immunosuppressive effect on the adaptive immune response promoting the development of regulatory T cells^16, 17^. We therefore further assessed the ability of 1,25(OH)_2_D_3_ vitamin D to induce a regulatory phenotype on CD4^+^CD25^−^ T cells after 5 days *in vitro* activation by aCD3 and CHO-CD80. All groups (AA, AG, GG) upregulated CTLA-4 and Foxp3 expression (Fig. 5) upon vitamin D stimulation. In untreated conditions (without vitamin D) cells from GG, AA and AG individuals showed similar induction of CD25 as indicated by the % of CD25 expressing cells [GG: 66% ± 14, AA: 64% ± 14 and AG: 62% ± 19 (mean value ± SD)] and CD25 MFI [GG:151 ± 114, AA: 195 ± 152, AG: 157 ± 131 (mean value ± SD)]. Stimulation with vitamin D did not alter the % of CD25 expressing cells but resulted in significant induction of CD25 MFI in GG (from 151 to 229 mean value) (*p = 0.018) and AA (from 195 to 251 mean value) (*p = 0.016), thus causing a 1.5 and 1.3 fold induction, respectively. In AG, vitamin D induced a lower CD25 expression (from 157 to 191 mean value) (Fig. 5). It is known that CD25 expression is upregulated by TCR stimulation and CD28, as well as by vitamin D via CD28 signaling^14^. In our experiments vitamin D induced CD28 expression and IL-2 cytokine secretion at similar levels across genotypes (Supplementary Figures 2 and 3). It is also well known that high CD25 expression defines T regulatory cells, thus higher CD25 expression could possibly mean higher frequency of T regulatory cells. Our data however on Treg frequencies based on CD25 expression as well as Foxp3 and CTLA-4 expression revealed an induction of regulatory cells after vitamin D stimulation with no significant differences across genotypes (Fig. 6). CD25 is considered an activation marker, thus the higher expression on T cells from GG and AA may mean a more activated phenotype.

Treg cell induction was further assessed based on the co-expression of CD25, Foxp3 and CTLA-4. Tregs were therefore defined as CD25^+^ Foxp3^hi^ CTLA4^+^. Our data show a similar frequency of Tregs across genotypes in basal conditions (VitD−) [GG: median = 11% range (4.2–21.7), AA: median = 11% range (3.7–29.0), AG: median = 9% (range: 5.6–23.6); values show the proportion of CD25^+^ cells expressing Foxp3 and CTLA4]. The presence of vitamin D (VitD+) significantly increased the proportion of Foxp3^hi^ CTLA4^+^ cells in the CD25^+^ population in each group [GG: median = 25% (range: 5.0–43.8), AA: median = 25% (range: 7.7–43.7), AG: median = 20% (range: 6.7–46.2)] (Fig. 6A,B). No significant differences were detected in the proportion of induced Tregs in the presence of vitamin D across genotypes (Fig. 6A,B). The median fluorescence intensity of Foxp3 and CTLA-4 molecules was similar across genotypes in the presence and absence of vitamin D (Fig. 6C,D).

**Figure 7:**
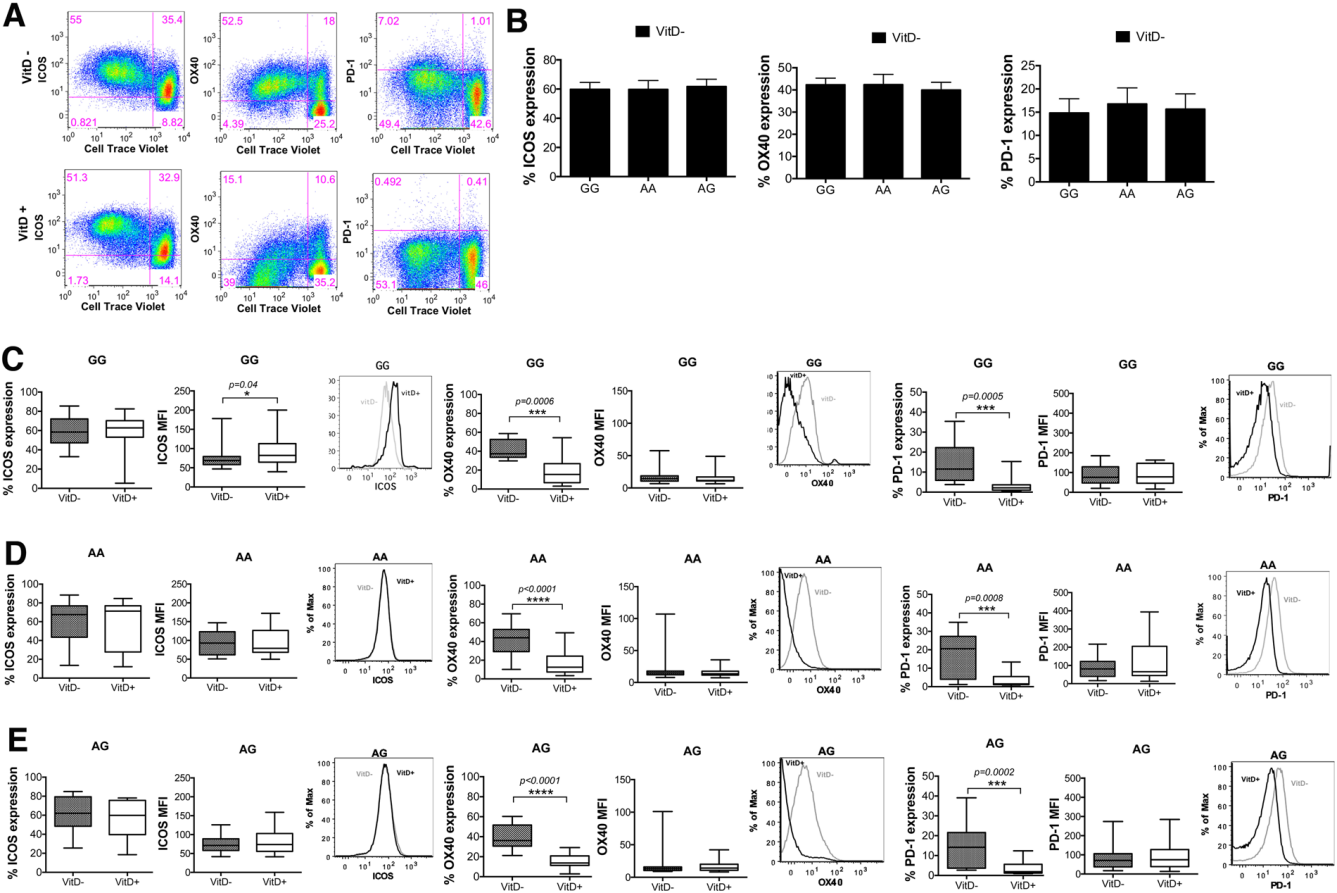
AA, AG and GG individuals express similar levels of ICOS, OX40 and PD-1 molecules after T cell activation. CD4^+^CD25^−^ T cells were activated *in vitro* with aCD3 antibody and CHO-CD80 cell line in the presence or absence of 1,25(OH)_2_D_3_ for 5 days. (**A**) Representative flow cytometry plots showing proliferating T cells expressing ICOS, OX40 and PD-1. (**B**) Percentage of proliferating cells that express ICOS, OX40 and PD-1 under no vitamin D treatment conditions (VitD−). (**C–E**) Data show the percentage of proliferating cells that express ICOS, OX40 and PD-1 and their respective median fluorescence intensity values for GG, AA and AG genotypes of the PSC CD28 risk allele locus. Representative overlay histograms showing the expression of each marker in the presence and absence of 1,25(OH)_2_D_3_. *p < 0.05, ***p < 0.001, ****p < 0.0001 using Wilcoxon matched-pairs signed rank test.

All groups (AA, AG, GG) downregulated OX40 and PD-1, with no significant differences across the groups being detected. The expression of ICOS activation marker on CD4^+^CD25^−^ T cells after *in vitro* stimulation with anti-CD3 antibody and CHO-CD80 cell line was similar across genotypes albeit slightly lower in GG and AG compared to AA individuals [MFI in GG: 78 ± 35, AA: 94 ± 33 and AG: 76 ± 24 (mean value ± SD)]. In GG donors stimulation with 1,25(OH)_2_D_3_ induced ICOS MFI [from 78 ± 35 to 96 ± 48 (mean value ± SD) by 1.2 fold (*p* = 0.042)]. In AA there was no change in ICOS MFI after stimulation with vitamin D and in AG there was only minimal ICOS induction (Fig. 7). The significant upregulation of ICOS MFI on T cells from GG donors after vitamin D stimulation is possibly because there was lower ICOS expression in untreated conditions as a starting point thus the difference in its expression was seen more robust after vitamin D stimulation. It is known that ICOS expression requires TCR activation and CD28 co-stimulation. We and others have shown that vitamin D can increase the median fluorescence of CD28 on CD4^+^ T cells^3, 18^, and our current data show an induction of CD28 MFI after vitamin D stimulation at similar levels across genotypes (Supplementary Figure 3)], thus the differential level of ICOS MFI on CD4^+^ T cells across genotypes seems not to be attributed to effects of vitamin D and CD28 expression. Notably, vitamin D receptor (VDR) binding regions have been detected closer to CD28 and CTLA-4 genes (one close to the promoter region of CD28, two in between CD28 and CTLA-4, and one in the CTLA-4 gene itself). In either side of ICOS gene there have been detected some VDR binding sites but they are more distant, therefore CD28 and CTLA-4 are more likely targets of direct regulation by vitamin D than is ICOS^19^. ICOS plays an important role in the development and effector functions of Th1, Th2, Th17 and Treg cells^20^, thus the higher expression of ICOS on GG donors may suggest differential effects on immune cell activation after its stimulation. Therefore, these data further verify the immunoregulatory role of vitamin D and the equal responsiveness of T cells across different genotypes to 1,25(OH)_2_D_3._

**Figure 8:**
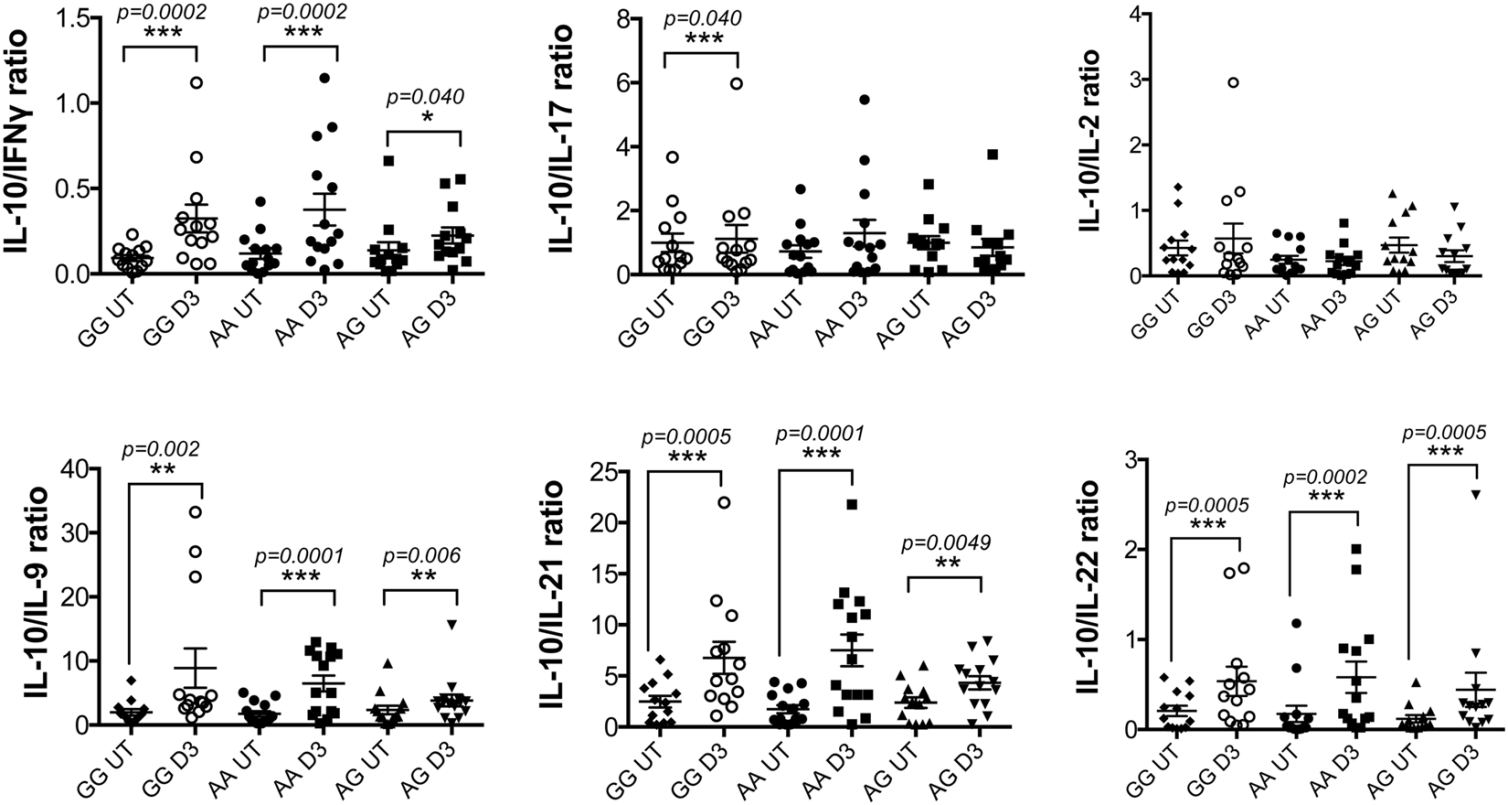
AA, AG and GG individuals respond to vitamin D and produce regulatory cytokines. CD4^+^CD25^−^ T cells were activated *in vitro* with aCD3 antibody and CHO-CD80 cell line in the presence or absence of 1,25(OH)_2_D_3_. At day 3, cell-free supernatant was collected and tested for the presence of secreted cytokines. Data show the ratio of IL-10 versus pro-inflammatory cytokines IFNγ, IL-17, IL-2, IL-9, IL-21 and IL-22. **p < 0.01, ***p < 0.001 using Wilcoxon matched-pairs signed rank test.

The level and type of co-stimulation that T cells receive plays an important role in determining their differentiation and cytokine production^21^. Therefore, we also assessed the effect of CD28 genotype upon CD4^+^ T cell cytokine production. Genotype had no effect upon cytokine production in the absence of 1,25(OH)_2_D_3_ and T cells from all three genotypes were able to respond to 1,25(OH)_2_D_3_ as indicated by the increased ratio of anti-inflammatory IL-10 versus the pro-inflammatory cytokines IFNγ, IL-17, IL-9, IL-21 and IL-22 (Fig. 8, Supplementary Figure 2). Therefore, these data also indicate an equivalent ability of each CD28 genotype to transmit the co-stimulation signal to respond to 1,25(OH)_2_D_3_.

## Discussion

Studies on the biological significance of identified genetic susceptible loci are limited. Many polymorphisms linked to human autoimmune and immune-mediated diseases have been identified through genome-wide association studies (GWAS), however the functional relevance of most of these genetic variations remain undefined. Here we explored the association of the confirmed PSC disease risk allele rs7426056 in the *CD28* locus with expression of CD28 and T cell function and responsiveness to variable stimuli in healthy individuals. We confirm that: (i) basally there is genotype dependent effect on gene expression in carriers of the susceptible CD28 variant; (ii) an interplay between viral infection and genotype might be also important for CD28 expression; (iii) signaling of CD28 is not different across the genotypes and neither is their response to vitamin D; and (iv) there is a genotype effect on response to TNFα inflammatory stimuli, which can be overcome by vitamin D.

The rs7426056 CD28 genetic variant is located 3.5 kb downstream of the *CD28* 3′UTR and approximately 120 kb upstream of *CTLA4*. It is in almost complete linkage disequilibrium (r^2^ = 0.99) (https://caprica.genetics.kcl.ac.uk/~ilori/ld_calculator.php) with the genetic variant rs1980422, also within the CD28 locus, that has been associated with rheumatoid arthritis locus^4^. The large majority of disease-associated SNPs are located in between genes and such non-coding SNPs may lead to decreased or enhanced production of the transcript and/or its translation into protein by affecting enhancers, microRNAs, or through long-range transcription regulation^22^. Our data show an altered gene expression in carriers of the susceptible CD28 variant, however no differences in protein levels were detected, at least on CD4^+^ T cells that were studied. A significant correlation between another CD28 variant (rs6435203), which is in close linkage disequilibrium with the studied rs7426056 and *CD28* mRNA expression has been also reported^7^. The triggers of expansion of CD28^−^ T cells are unclear; continuous antigenic stimulation^23^ and *in vitro* replicative senescence^24^ have been reported to influence CD28 expression. An association between *HLA-DRA* and loss of CD28 expression on memory CD8^+^ T cells has been reported in healthy subjects^25^ therefore a combination of genetic and environmental factors may further contribute to loss of CD28 expression. In addition, our data show that the signaling of CD28 and specifically the ability of CD4^+^ T cells to respond to vitamin D is not different across genotypes as indicated by the induction of a regulatory phenotype and an increased anti-inflammatory/pro-inflammatory cytokine ratio in vitamin D treated samples.

Identifying the functional significance of GWAS-identified disease-associated variants is now a major but important challenge as it will allow enhanced understanding of disease pathologies from which novel preventative and therapeutic strategies can be developed. Overall, our study into the functional significance of the PSC-associated CD28 variant, rs7426056, has identified a possible inhibitory effect of the SNP upon CD28 mRNA expression, although CD28 protein *ex vivo* remained unchanged in CD4^+^ T cells and we observed a protective effect of the variant upon CD28 loss under inflammatory conditions. To note, our system used a mixed CD4 population consisting of both naïve and memory cells thus unable to distinguish between polarization of naïve cells and reactivation of memory cells. However, a mixture of CD4 populations is expected to be present *in vivo*, thus we believe our approach was a more realistic approach. The mixture of naïve and memory cells was similar across genotypes, thus this should not interfere with the validity of findings.

The functional effect of this CD28 SNP in PSC pathology therefore remains slightly unclear. It is likely that rs7426056, is not an independent regulator of CD28-mediated T cell responses but becomes significant in PSC when inherited with other PSC-associated gene variants. Our study does not suggest an interaction of rs7426056 with the environmental variant vitamin D but other PSC-associated environmental risk factors could affect its biological effect. One interesting regulator of immune responses that is gaining much attention as a regulator of immune responses, is metabolism. Notably, CD28 co-stimulation has been shown to enhance the expression of glucose transporters, glucose uptake, and glycolysis in human T cells in a PI3K dependent manner^26^. Whether rs7426056 affects T cell metabolism is therefore of future interest.

An alternative explanation for the association of rs7426056 with PSC is its co-inheritance with a functional disease variant by linkage disequilibrium. Indeed, it is very likely that many GWAS-identified disease variants are essentially tags for a limited number of functional disease-causing variants. Studies to identify the roles of GWAS variants should therefore focus initially on those in or surrounding genes for which a functional involvement of the encoded factor has already been demonstrated. However, as our study suggests, multiple genetic and environmental factors could affect the functional manifestation of the mutation and its involvement in disease. Understanding the potential role of genetic association signals in disease biology will therefore require complex and carefully controlled studies. To date, genome wide association studies in PSC have identified 23 susceptibility loci and this is mirrored by even more risk loci for other autoimmune diseases. Therefore, in this context, whilst of course ultimately one wishes to study gene effects in patients (and more specifically in target tissues and target cell types, controlled for risk, stage and disease treatment), it is scientifically logical to start evaluating a gene risk locus in healthy controls: this allows investigators to attempt to model the impact of the genetic variation more closely, without all the confounding effects of multiple other disease risk gene effects, the consequences of disease and its progression, as well as the impact of therapies.

A methodological constraint of our study and one that makes the task of analyzing healthy individuals in which a single variant of interest is being studied, is the power calculation analysis. Initial power calculation analysis based on our previous findings^3^ revealed that to detect a difference of 50% between the control and vitamin D treated groups a sample size of 14 patients per group would be sufficient to detect a difference of this size with alpha = 0.05 and power of 80%. Based on the sample size we have used (n = 14 per group of healthy individuals) one-way ANOVA comparing the logged values of CD4^+^CD28^−^ T cell frequencies showed a 6.7-fold minimal detectable difference between the groups at 80% power and 5% alpha. Our sample size was limited however by substantial ethical and logistic practicalities (i.e. need for same day shipping of blood in working hours, day of the week the bleeding should take place on, availability and willingness of subjects to take part, and season of the year [acquisition and processing of 42 samples in total lasted 5 months]) and therefore it is acknowledged that our study power could in the ideal world have been augmented, and future much larger studies overcoming the logistics of working with fresh samples from large numbers of healthy volunteers should account for this. Our main genotype difference is between GG vs AG/AA, at around 2.5-fold (geometric mean: 0.35 vs 0.88/0.81), and for this to be statistically significant confirmed finding, a much larger number of healthy individuals (approaching 200 in total across genotypes) would be needed, something we could not overcome in this study.

To conclude, our study is one of the very few that attempts to address the biological significance of CD28 genetic variant in the pathology of an immune-mediated liver disease of unknown etiology. Understanding the effects of the different genotypes on gene expression, susceptibility to inflammatory stimuli and environmental factors has started to be the focus of several research attempts. Therefore, hopefully extension of such studies as ours will lead to better understanding of how genetic risk loci associated with disease impact meaningful biologic pathways.

## Methods

### Study population

Peripheral blood from healthy individuals with known risk (AA and AG) and control genotypes (GG) (n = 14 per group) was collected via the Cambridge NIHR BioResource. All samples were obtained with Local Research and Ethics Committee approval and informed patient consent. All experimental protocols were approved by NHS Health Research Authority and NRES Committee West Midlands - South Birmingham (REC 2003/242) and all methods were carried out in accordance with relevant guidelines and regulations.

Table 1 illustrates the characteristics (sex and age) of all individuals included in the study. All data were assessed blinded to genotype.

**Table 1:** Characteristics of all individuals included in the study.

Donor ID	Sex	Age (years)	Category
# 01	F	49	AA
# 02	F	51	AG
# 03	F	43	GG
# 04	F	33	AG
# 05	F	32	GG
# 06	F	39	AA
# 07	F	48	AA
# 08	F	47	AG
# 09	F	48	GG
# 10	M	47	GG
# 11	M	45	AG
# 12	M	50	AA
# 13	M	51	AA
# 14	M	49	AG
# 15	M	52	GG
# 16	F	47	AG
# 17	F	41	GG
# 18	F	47	AA
# 19	F	52	GG
# 20	F	47	AA
# 21	F	48	AG
# 22	F	53	AG
# 23	F	43	GG
# 24	F	51	AA
# 25	F	52	AG
# 26	F	52	AA
# 27	F	47	GG
# 28	F	48	GG
# 29	F	43	AG
# 30	F	45	AA
# 31	F	35	GG
# 32	F	34	AG
# 33	F	37	AA
# 34	M	50	AA
# 35	M	53	GG
# 36	M	46	AG
# 37	F	51	GG
# 38	F	57	AA
# 39	F	48	AG
# 40	M	42	AA
# 41	M	50	GG
# 42	M	41	AG

### Cell Isolation

Peripheral blood mononuclear cells (PBMCs) were isolated by density gradient centrifugation using Lympholyte (Cedarlane Laboratories, Burlington, Canada) for 20 min at 800 g. CD4^+^CD25^−^ T cells were purified from PBMCs using a custom made negative selection antibody cocktail (StemCell Technologies), according to manufacturer’s instructions.

### Cell Culture

Chinese hamster ovary (CHO) cells expressing CD80, were cultured in DMEM (Life Technologies, Paisley, UK) supplemented with 10% v/v FBS (Life Technologies), 1% penicillin, streptomycin (Life Technologies) and incubated at 37 °C in a humidified atmosphere of 5% CO_2_.

### CD4^+^ CD25^−^ T cell *in vitro* stimulation

CD4^+^CD25^−^ T cells, enriched (on average) to >94% were cultured in RPMI1640 media supplemented with 1% penicillin-streptomycin-glutamine (PSG) and 10% foetal calf serum (FCS) (Life Technologies). For some studies as described, T cells were stimulated with aCD3/aCD28 Dynabeads (1 μl/well (~1:10 bead:cell); Life Technologies), IL-2 (50U/ml; Peprotech) in the presence/absence of TNFα (10ng/ml; Peprotech) with or without vitamin D (1,25(OH)_2_D_3_ 10 nM); ENZO Life Sciences). At 4-days, beads were removed using a magnet and cells were split to 0.5 × 10^6^ cells/ml. At day 6 cells were re-activated with aCD3/aCD28 beads for another cycle of activation for 4 days. At day 10 beads were removed, cultures were assessed and maintained at 0.5–1 × 10^6^ cells/ml. After that, cultures were assessed every 4 days and maintained at 0.5–1 × 10^6^ cells/ml. Cytokines and 1,25(OH)_2_D_3_ were also re-supplemented at the time of cell culture assessment. The concentration of IL-2 was increased to 100U/ml after 1 week. Cells were cultured for up to 21 days. CD28 expression was assessed at days 14 and 21 using flow cytometry.

For separate experiments, CD4^+^CD25^−^ T cells were labelled with cell trace violet (Life Technologies) per manufacturer’s instructions. CHO-CD80 cells were trypsinized and fixed with 0.025% glutaraldehyde for 3 mins at room temperature followed by quenching with media containing FCS. Fixed cells were washed and finally resuspended in RPMI 1640 + 10% FCS + 1% PSG. Cell trace violet-labelled T cells (0.5 × 10^6^ cells/ml) were co-cultured with CHO-CD80 (at 5:1 ratio) with anti-CD3 (OKT3, at 0.5 μg/ml), with or without 10 nM 1,25(OH)_2_D_3_ (ENZO Life Sciences) in 96-U plates for 5 days. Cell free supernatant was collected at day 3 for assessment of cytokine expression.

### Luminex cytokine analysis

Cell free supernatant from T cell-CHO-CD80 day 3 co-cultures was collected and assessed for cytokine expression using the human Th1/Th2/Th17/Th22/Treg Procartaplex Multiplex Immunoassay (eBiosciences catalog number EPX180-12165-901), according to manufacturer’s instructions. The cytokines measured were: GM-CSF, IFNγ, IL-1, IL-12, IL-13, IL-18, IL-2, IL-5, IL-6, TNFα, IL-4, IL-10, IL-17, IL-21, IL-22, IL-23, IL-27, IL-9.

### Flow Cytometry

Flow cytometric analysis was performed on peripheral blood T cells using a Cyan flow cytometer (Beckman Coulter, Bucks, UK), and analysed using FlowJo (version 9). CD28 expression on unstimulated PBMCs (at day of isolation) was studied by surface staining. Cultured cells were collected (at days 14 and 21) and stained with live/dead marker (Zombie, Biolegend) for 20 minutes at room temperature, followed by washes and surface staining with CD3-FITC (clone HIT3a), CD4-APC (clone RPA-T4) and CD28-PE (clone 28.2) (all from BD Biosciences). After 5-days T cell – CHO-CD80 co-culture, cells were stained with: CD4-PerCP-Cy5.5 (clone RPA-T4, BD Biosciences), CD28-PeCY7 (clone 28.2, Biolegend), ICOS-FITC (clone REA192, Miltenyi Biotec), OX40-PE (clone ACT35, BD Biosciences) and PD-1-APC (clone MIH4, BD Biosciences).

For intracellular and nuclear protein detection cells were stained for live/dead marker prior to fixation and permeabilization using the Foxp3/transcription factor staining buffer set according to manufacturer’s instructions. After fixation and permeabilization cells were stained with: CD4-PerCP-Cy5.5 (clone RPA-T4, BD Biosciences), CD28-PeCY7 (clone 28.2, Biolegend), CD25-BB515 (clone 2A3, BD Biosciences), CTLA-4 (clone BNI3, BD Pharmingen) and Foxp3-APC (clone PCH101, eBioscience). Analysis of activation markers (CD25, PD-1, ICOS, CTLA-4, OX40) and transcription factors (Foxp3) was focused on activated/proliferating cells as inclusion of non-activated cells would have skewed the results.

### Analysis of CMV positivity in plasma samples

Plasma samples were used to determine CMV IgG titres. A CMV IgG ELISA assay developed at the University of Birmingham by Professor Paul Moss’s group was used. A sample from a known CMV seropositive donor (positive control) and a known CMV seronegative donor (negative control) for which multiple aliquots were frozen was run with every single plate in order to document assay precision and reproducibility. All samples were run in duplicate in CMV lysate coated wells, as well as mock lysate coated wells, and the average absorbance value seen in the mock lysate coated wells (background non-specific binding) was subtracted from the average absorbance value in the CMV lysate coated plate.

### Quantification of mRNA expression levels by RT-PCR

Total RNA was extracted from uncultured peripheral blood mononuclear cells using the RNeasy mini kit (Qiagen, UK) according to manufacturer’s instructions. The eluted RNA concentration was measured using a NanoDrop Spectrophotometer (Thermo Fisher Scientific). 50 μg of extracted RNA was transcribed into cDNA using iScript cDNA synthesis kit (BioRad, Hercules, CA). Quantitative analysis of CD28 mRNA expression was performed using Taqman Fluorogenic 5′ nuclease assays using gene-specific 5′FAM labelled probes (Hs01007422_m1, Life Technologies) run on ABI 7900 sequencer with beta-actin used as endogenous control (Hs01060665_g1, Life Technologies). Values are represented as the difference in Ct values normalized to β-actin for each sample as per the following formula: Relative RNA expression = 2^−ΔCt^ where ΔCt = Ct of CD28 – Ct of β-actin^27^.

### Statistical Analyses

Statistical analyses were performed using GraphPad Prism. Data not normally distributed were evaluated using Wilcoxon matched-pairs signed rank test and Kruskal-Wallis test. Values of *P* < 0.05 were considered significant.

### Data Availability

All data generated or analysed during this study are included in this published article (and its Supplementary Information files).

## Electronic supplementary material

Supplementary Information
