# Supplementary material for: Genetic variation at the CD28 locus and its impact on expansion of pro-inflammatory CD28 negative T cells in healthy individuals
Source: Sci Rep. 2017 Aug 9;7:7652. doi: 10.1038/s41598-017-07967-2 (PMC5550460; doi:10.1038/s41598-017-07967-2)
Supplement: Supplementary file 1 — Supplementary Information [file 41598_2017_7967_MOESM1_ESM.pdf]

Genetic variation at the CD28 locus and its impact on expansion of pro-inflammatory CD28 negative T cells in healthy individuals

Evaggelia Liaskou[1], Louisa Jeffery[2], Dimitrios Chanouzas[3], Blagoje Soskic[4], Michael F Seldin[5,6], Lorraine Harper[3], David Sansom[4], Gideon M Hirschfield[1*]

[1] Centre for Liver Research and NIHR Birmingham Biomedical Research Unit, Institute of Immunology and Immunotherapy, University of Birmingham, Birmingham, UK

[2] Institute of Metabolism and Systems Research, College of Medical and Dental Sciences, University of Birmingham, Birmingham, UK

[3] Institute of Inflammation and Ageing, University of Birmingham, Birmingham, UK

[4] Institute of Immunity and Transplantation, University College London and Royal Free Hospital, London NW3 2PF, UK

[5] Department of Biochemistry and Molecular Medicine, University of California at Davis, Davis, CA 95616, USA

[6] Division of Rheumatology, Allergy and Clinical Immunology, University of California at Davis School of Medicine, Genome and Biomedical Sciences Facility, 451 Health Sciences Drive, Suite 6510, Davis, CA 95616, USA

Supplementary Information

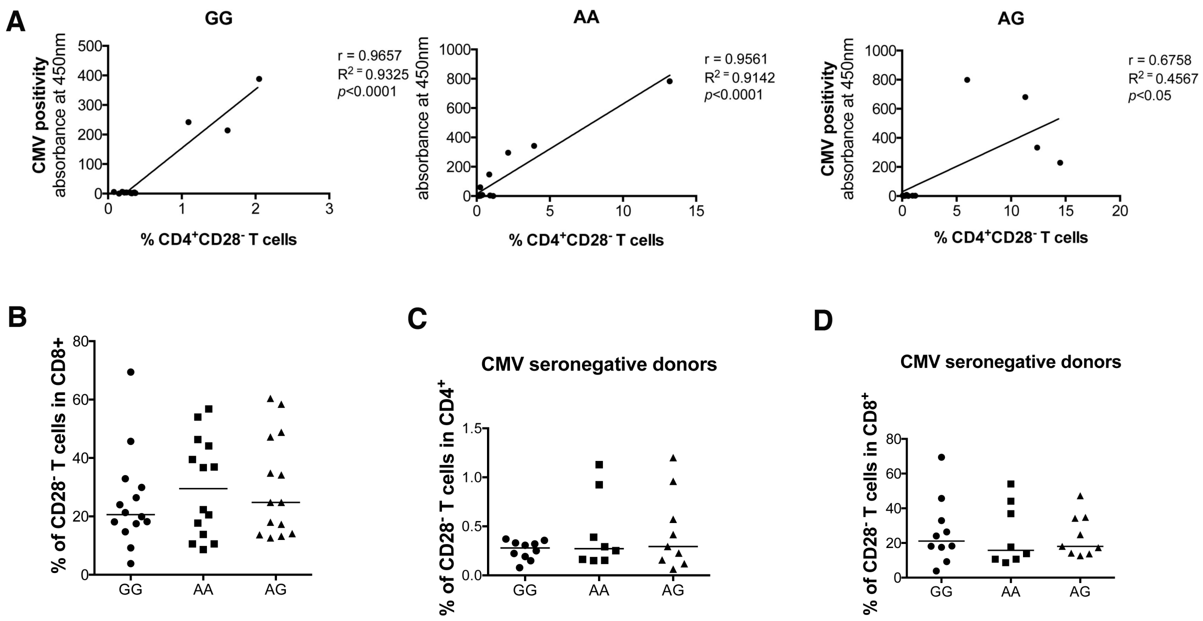

Supplementary Figure 1. High frequency of CD4$^+$CD28$^-$ T cells is associated with CMV positivity. (A) Plasma samples from GG, AA and AG individuals were tested for CMV positivity using ELISA. CMV IgG titres (CMV positivity) are plotted against frequency of CD4$^+$CD28$^-$ T cells. **(B)** Data show the proportion of CD8$^+$ T cells that have lost CD28 expression in total peripheral blood mononuclear cells (PBMCs). Line indicates median value. **(C, D)** Data show the proportion of CD28$^-$ T cells in CD4 and CD8 T cell subsets after exclusion of CMV seropositive donors.

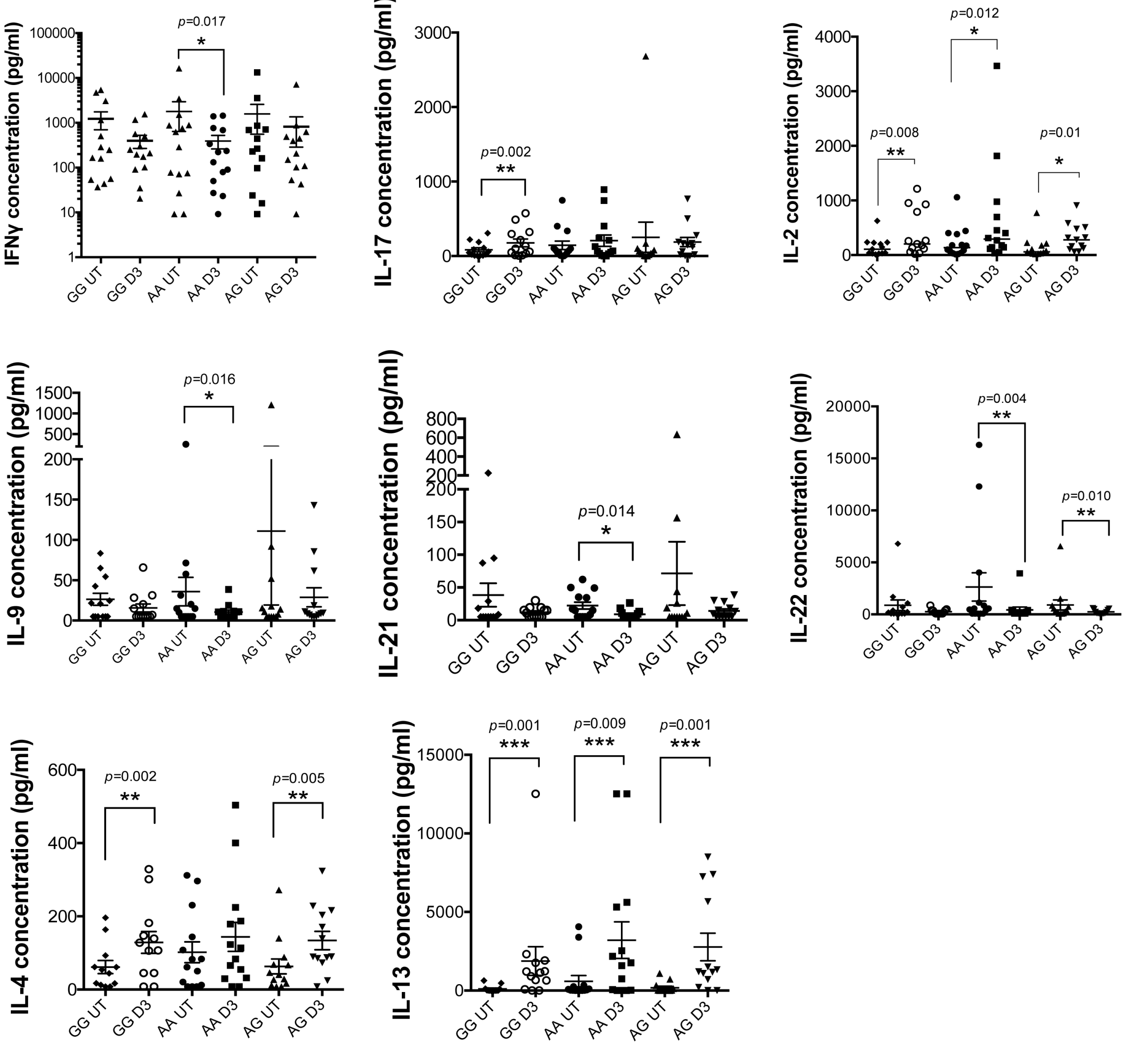

Supplementary Figure 2. AA, AG and GG individuals respond to vitamin D and produce regulatory cytokines. $CD4^{+}CD25^{-}$ T cells were activated in vitro with aCD3 antibody and CHO-CD80 cell line in the presence or absence of $1,25(OH)_2D_3$. At day 3, cell free supernatant was collected and tested for the presence of secreted cytokines. Statistical significance was tested using Wilcoxon matched-pairs signed rank test.

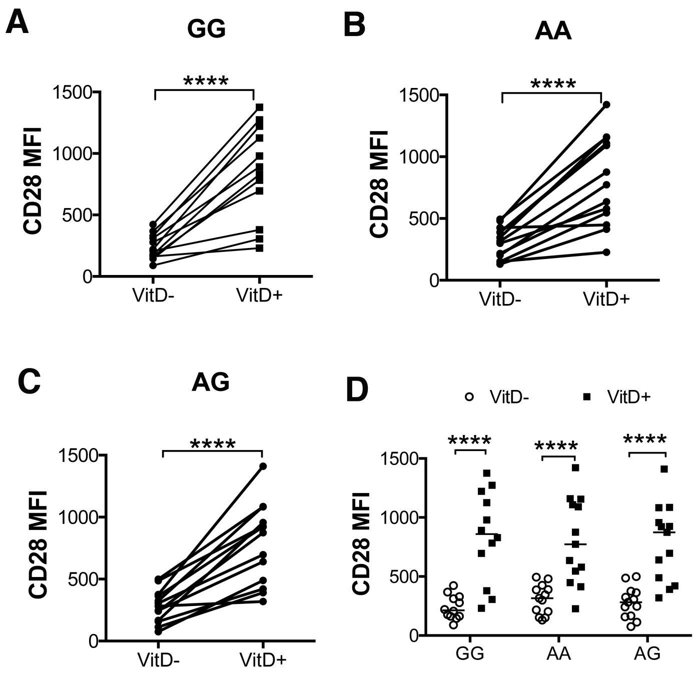

Supplementary Figure 3. AA, AG and GG individuals respond to vitamin D and upregulation CD28 expression. $CD4^{+}CD25^{-}$ T cells were activated in vitro with aCD3 antibody and CHO-CD80 cell line in the presence or absence of $1{,}25(OH)_2D_3$. $CD4^{+}CD25^{-}$ T cells were activated in vitro with aCD3 antibody and CHO-CD80 cell line in the presence or absence of $1{,}25(OH)_2D_3$ for 5 days. (A-C) Data show CD28 MFI across genotypes. (D) Collective data from all donor samples. Lines indicate median values ***p<0.001 using Wilcoxon matched-pairs signed rank test.
